# Acute Fatigue Responses to Occupational Training in Military Personnel: A Systematic Review and Meta-Analysis

**DOI:** 10.1093/milmed/usac144

**Published:** 2022-05-27

**Authors:** Brian Heilbronn, Kenji Doma, Wade Sinclair, Jonathan Connor, Lachlan Irvine-Brown, Anthony Leicht

**Affiliations:** Royal Australian Army Medical CORPS, Australian Army, Australian Defence Force, Australia; Sport and Exercise Science, James Cook University, Townsville, QLD 4811, Australia; Sport and Exercise Science, James Cook University, Townsville, QLD 4811, Australia; Sport and Exercise Science, James Cook University, Townsville, QLD 4811, Australia; Sport and Exercise Science, James Cook University, Townsville, QLD 4811, Australia; Royal Australian Army Medical CORPS, Australian Army, Australian Defence Force, Australia; Sport and Exercise Science, James Cook University, Townsville, QLD 4811, Australia; Australian Institute of Tropical Health & Medicine, James Cook University, Townsville, QLD 4811, Australia

## Abstract

**Introduction:**

Military personnel are required to undertake rigorous physical training to meet the unique demands of combat, often leading to high levels of physiological stress. Inappropriate recovery periods with these high levels of physical stress may result in sub-optimal training and increased risk of injury in military personnel. However, no reviews have attempted to examine the magnitude of training-induced stress following military training activities. The aim of this systematic review was to assess the magnitude of physiological stress (physical, hormonal, and immunological) following task-specific training activities in military personnel.

**Methods:**

An extensive literature search was conducted within CINAHL, PubMed, Scopus, SportDiscus, and Web of Science databases with 7,220 records extracted and a total of 14 studies eligible for inclusion and evaluation. Study appraisal was conducted using the Kmet scale. Meta-analysis was conducted via forest plots, with standard mean difference (SMD, effect size) and inter-trial heterogeneity (*I*^2^) calculated between before (preactivity) and after (12–96 hours postactivity) military-specific activities for biomarkers of physiological stress (muscle damage, inflammation, and hormonal) and physical performance (muscular strength and power).

**Results:**

Military training activities resulted in significant levels of muscle damage (SMD = −1.28; *P *= .003) and significant impairments in strength and power (SMD = 0.91; *P *= .008) and testosterone levels (SMD = 1.48; *P *= .05) up to 96 hours postactivity. There were no significant differences in inflammation (SMD = −0.70; *P *= .11), cortisol (SMD = −0.18; *P *= .81), or insulin-like growth factor 1 (SMD = 0.65; *P *= .07) when compared to preactivity measures.

**Conclusions:**

These findings indicate that assessments of muscle damage, anabolic hormones like testosterone, strength, and power are effective for determining the level of acute stress following military-specific activities. With regular monitoring of these measures, appropriate recovery periods may be implemented to optimize training adaptations and occupational performance, with minimal adverse training responses in military personnel.

## INTRODUCTION

The physiological demands of military training impose unique physical stresses not generally experienced in typical civilian occupations.^1^ Military training involves a vast spectrum of activities from equipment maintenance, physical fitness training, rehearsals of tactics, training, and procedures^2^ through to live fire range practices.^3^ Thus, military personal must undertake specific physical preparation training to meet the demands of these activities.^4^ Physical preparation within military organizations can be generally categorized into 3 different training modalities: (1) physical training (PT); (2) military skills training (MST); and (3) field training exercises (FTX). Physical training follows a traditional approach to fitness enhancement in a controlled environment (e.g., gymnasium) and provides the underlying physical capacity to undertake strenuous occupational tasks encountered in the military.^4,5^ Military skills training incorporates the practical application and rehearsal of fundamental warfighting tactics in a barracks or controlled field environment.^6^ Field training exercises are capstone activities where units work in a complete tactical setting and undertake various missions against an opposing force.^3^ While MST and FTX are not specifically forms of traditional PT, they often require tactical personnel to carry substantial loads of up to 45 kg,^7^ resulting in an increased metabolic demand^8^ and potential increased risk of injury.^5,9^ Furthermore, these activities can span hours through to larger-scale training exercises lasting several weeks.^1,10^ These unique occupational demands require appropriate physical preparation with all 3 modalities essential for military personnel to undertake their strenuous occupational tasks.^4,5^

Investigations of the physiological effects of MST, PT, and FTX have identified that significant levels of fatigue can be induced as a result of these training modalities.^11–13^ The training-induced stress from MST, PT, and FTX can take several days to recover from, resulting in temporary declines in the ability to complete subsequent military-specific tasks and compromising combat effectiveness.^1–3,12–15^ This stress has been identified in physically active populations via biomarkers of muscle damage (e.g., creatine kinase [CK]), inflammation (e.g., C-reactive protein [CRP]), hormonal responses (e.g., cortisol [CORT]), and physical performance measures (e.g., counter-movement jump [CMJ]).^12,15–17^ However, changes in these measures following military training have been variable. For example, Hamarsland et al.^12^ reported reductions in lower body strength and testosterone (TEST) levels, and an increase in CORT, after the completion of 1 week of arduous military training. In contrast, Taipale et al.^18^ reported no changes in these variables at 18 hours after 50 minutes of loaded marching. Furthermore, others^6,13,15^ demonstrated elevations in TEST during a 12-week training cycle and/or 72 to 96 hours after an FTX. While these inconsistencies in stress responses may be due to different methodological designs, collectively these reports make it difficult to fully comprehend the influence of occupational and training tasks on recovery periods for military personnel. For example, the intervention time frames examined ranged from 60 seconds^19^ to 12 weeks.^13^ The physical components of each intervention were also variable and ranged from one-off, maximal attempts of occupational tasks (e.g., a stretcher carry)^20^ to survival, evasion, resistance and escape training (SERE) lasting up to 2 weeks^21^ and to full FTX activities, covering the full spectrum of soldier’s occupational tasks.^3^ Furthermore, the occupational experience of these populations was diverse with soldiers examined at different stages of their careers and therefore different levels of training proficiency. For example, Koury et al.^22^ examined army cadets who were at the earlier stages of their career, whereas Szivak et al.^21^ examined soldiers with greater than 3 years of experience. Therefore, a systematic exploration of the relevant literature, including various mechanisms such as biomarkers and physical performance measures, may provide a clearer understanding of the impact of these activities on soldier responses for enhanced occupational performance.

To date, previous reviews have examined the acute effects of sustained operations,^8^ compared military findings on functional overreaching, nonfunctional overreaching and overtraining syndrome,^23^ and summarized the current understanding of key physiological biomarkers of physiological stress and their underlying mechanisms before, during, and after military training.^24^ Collectively, these review papers identified that military personnel exhibited serious physiological impairments^8^ and functional and nonfunctional overreaching as a result of military training^23^ and suggest a balanced biomarker panel may be useful to monitor training-induced impairments in military personnel.^24^ Such training-induced impairments could have long-lasting and catastrophic effects in an operational setting for soldiers, including increased exposure to enemy fire,^25^ reduced accuracy of weapons fire, hampered ability to effectively engage the enemy, and subsequently compromised survivability and lethality.^26^ Impairments in physical capability caused by acute physiological stress from occupational tasks may compromise training quality and subsequently lead to sub-optimal physical and physiological adaptations,^27^ as well as an increased risk of injury.^28^ While important to understand the acute impact of military training on performance,^8,23,24^ the aforementioned reviews did not summarize the magnitude of training-induced stress during recovery following military-specific training (i.e., 12–96 hours postactivity). This is an essential follow-up period after training, given that training sessions are generally separated by 24 to 48 hours of recovery to optimize strength, power, and aerobic development in military personnel.^4^ Undertaking training during this between-training sessions period may result in sub-optimal adaptations or could lead to instances of overtraining.^23^ Furthermore, future work to understand soldier’s general responses to, and recovery from, military-specific training to potentially minimize the risk of sub-optimal training and/or injuries is warranted.^23,24^ Therefore, the aim of this systematic review with meta-analysis was to examine the acute effects of various military-specific physical training (MST, PT, and FTX) on biomarkers of exercise-induced stress, muscular contractility, and physical performance measures. A greater understanding of these impacts would allow for better development and implementation of training methods to optimize training adaptations and military force capability.

## METHODS

The PRISMA guidelines^29^ were followed for the methodology and reporting of data in this systematic review and meta-analysis, following a PICO (population, intervention/exposure, comparison, and outcome) approach.

Studies were considered eligible and included in this review provided that they met the following PICO criteria:

1. *Population:* Military personnel or “tactical athletes” without injury that affected physical performance
2. *Intervention or exposure:* Studies employed a repeated measures design to examine the physiological effects of military-specific physical activities (e.g., loaded marching, stretcher carriage, and military field training).
3. *Comparison:* Studies compared outcomes prior to (baseline) and following military-specific physical activities
4. *Outcome:* Outcome measures included any biomarkers indicative of training-induced stress, such as muscle damage (e.g., CK and CRP), hormonal responses (e.g., CORT), and physical performance measures (e.g., CMJ, see *Outcome Measures* below).

Studies were excluded if: (1) they were reported in a language other than English; (2) no outcome measures were reported greater than 12 hours postactivity; or (3) they were reported as abstracts, reviews, or case reports.

The outcome measures for the current review included indicators of muscle damage, inflammation, and hormonal responses and changes in physical performance. These measures were previously reported as sensitive indicators to detect levels of physiological stress in athletes and tactical populations.^1,12,14,16,17^ Common biomarkers of muscle damage (e.g., CK, lactate dehydrogenase [LDH], and myoglobin [MGB]), inflammation (e.g., CRP, interleukin 1-8 [IL-1-8], and tumor necrosis factor alpha [TNF-a]), and hormonal responses (CORT, TEST, testosterone to cortisol ratio [T/C], Insulin-like growth factor one [IGF-1], triiodothyronine [T3], thyroxine [T4], thyroid-stimulating hormone [TSH], dehydroepiandrosterone [DHEA], dehydroepiandrosterone sulfate [DHEA-S], and sex hormone binding globulin [SHBG]) were examined. Physical performances indicative of muscular contractility were also regarded as indirect markers of muscle damage^30^ and included maximal voluntary isometric contractions for upper and lower body (e.g., leg and chest press, leg extension, and hand grip strength) and counter-movement and standing long jumps. Results of these tests were recorded by the height or distance achieved and/or maximal force produced. Previously, attenuated responses during these physical performance tests indicated the presence of residual fatigue.^17,31^ Outcome measures were extracted from included studies when reported at 12 to 96 hours following the completion of any military-specific physical activity, as this typified conventional rest periods experienced by soldiers following operational activities^8^ and peak time for exercise-induced changes in performance and biomarkers.^32^

A literature search up to June 29, 2021 was performed across 5 major electronic databases (CINAHL, PubMed, Scopus, SportDiscus, and Web of Science). For the PubMed search, 3 groupings of MeSH terms were utilized in combination (Table S1). Equivalent free text searches were conducted in the other databases (Table S1) with the reference lists of included studies screened as a supplementary search.

Abstract screening was conducted independently by 2 authors (BH and LIB) who actively served in the military in both infantry and physical performance development roles and were qualified Exercise Scientists (i.e., Bachelor’s degree). Abstracts were classified as meeting the inclusion criteria (i.e., yes), possibly meeting the criteria (i.e., maybe), or failing to meet the criteria (i.e., no). Inter-rater reliability was calculated from the review of a random sample (40%) of the total number of abstracts following screening. A weighted Kappa value of 0.85 (95% confidence interval: 0.76–0.94) was calculated and acceptable for inter-rater reliability.^33^ On completion of the screening process, the identified full-text articles were retrieved and further screened against the inclusion/exclusion criteria to obtain the final sample of studies (Fig. S1).

After full-text screening, information relating to study design, number of participants, participant demographics (Table S2), methodological design (e.g., study duration, physical activity, and timing of assessments), and main findings (Table S3) were compiled into customized forms. The preactivity and recovery (i.e., 12, 24, 48, 72, and 96 hours postactivity) values were then entered into a spreadsheet. A modified Kmet appraisal checklist was then used to critically appraise the methodological quality of the included studies for the internal validity of each intervention.^34^ Utilizing a 3-point ordinal scoring system (yes = 2, partial = 1, and no = 0) across 14 items, the Kmet provided a simultaneous assessment of the systematic, reproducible, and quantitative quality of research across a spectrum of study designs.^34^ As items 5 to 7 concerned the assessment of the random allocation of participants to treatment groups and blinding of participants and investigators, aspects deemed inapplicable due to the methodological design of included studies, these items were removed from the Kmet appraisal (i.e., best total score of 22). The individual Kmet scores were summed with the total score for each study converted into a percentage ([total score/22] × 100) with a score of >80% reflecting strong research quality, a score of 60% to 79% reflecting good research quality, a score of 50% to 59% reflecting adequate research quality, and a score <50% reflecting poor research quality.^35^ Kmet scoring was cross-checked by a second reviewer (JC), with a third reviewer (KD) approached to reach consensus, as required. A weighted Kappa value of 0.97 was calculated, which was acceptable for inter-rater reliability.^33^ Publication bias was examined by generating funnel plots using Review Manager Software 5 (RevMan, Version 5.3, Copenhagen: The Nordic Cochrane Centre, 2014). Given the requirement for military populations to be examined specifically for the current review, participant selection bias was unavoidable.

Review Manager Software 5 (RevMan, Version 5.3, Copenhagen: The Nordic Cochrane Centre, 2014) was utilized to conduct a meta-analysis along with the current systematic review. Outcome measures were included in the meta-analysis when reported by 4 or more studies.^36^ Biomarkers that were of the same constructs were combined into one forest plot to report on overall physiological response.^37^ Mean ± standard deviation was used to report all data from included studies with measures of dispersion originally reported as standard errors or confidence intervals converted to standard deviations.^38^ The included studies were assessed for heterogeneity among the samples utilizing an *I*2 statistic with values of 25%, 50%, and 75% classified as low, moderate, and high levels, respectively.^39^ Where data were reported as figures, corresponding authors were contacted for additional information that was then added to this review. When data were not provided, data were extracted from figures using digitizing software (WebPlotDigitizer, PLOTCON, USA). A random-effects model was employed to account for inter-study heterogeneity via forest plots, which was formulated by pooling the data from the included studies. Standardized mean differences (i.e., effect size) were calculated to determine the magnitude of preactivity vs. postactivity differences with values of 0.2, 0.5, and 0.8 classified as small, medium, and large, respectively.^40^ A *Z*-value formulated from the forest plot was also used to report the overall effect of the preactivity vs. postactivity comparisons. The level of exercise-induced physiological stress was interpreted based on statistical significance (*P *≤ .05) and standardized mean differences between preactivity and postactivity time points.

## RESULTS

A total of 6,599 abstracts from the databases were screened following the removal of duplicates (Fig. S1). Further screening of the abstracts led to the exclusion of most resulting in 44 full-text articles for review and 14 original articles included for critical appraisal and meta-analysis (Fig. S1).

A total of 431 participants were identified from the 14 included studies. The majority of participants were male (*n =* 416), with only one study reporting the inclusion of females (*n *= 15).^19^ The mean ranges for age, height, and mass were 19 to 27 years, 176 to 181 cm, and 72 to 85 kg for males and 29 ± 7 years, 167 ± 7 cm, and 65 ± 12 kg for females, respectively. These values indicated that the physical characteristics were similar between studies (Table S2). Study participants were recruited from a variety of military occupations and training schools, including regular military units,^2,3,6,15,19–21,41^ army recruit or cadet training,^22,42^ special forces selection courses,^12,43^ reservists,^18^ and SERE training^21,44^ (Table S2).

### Methodological Descriptions

#### Modality and duration of training

With respect to the stress-inducing activities of the 14 studies reviewed, 7 studies incorporated FTX-based activities only,^2,3,12,15,22,42,43^ while one included a short 4-day garrison training component,^6^ and another 2 studies investigated SERE training involving a 4-^21^ or 5-day^44^ didactic phase prior to a field phase (Table S3). The remaining 4 studies examined short duration (≤90 minutes) interventions including loaded marching on a treadmill^18,41^ or stretcher carriage while walking on a treadmill.^19,20^ Duration of all 14 studies ranged from 1 minute^19^ to 3 weeks.^2,3,15^

#### Recovery periods

Five studies reported outcome measures across multiple, postactivity time points (12–96 hours),^12,19,22,41,43^ whilst the remaining 9 studies reported outcome measures at 1 postactivity time point only. Three studies reported outcome measures at 24 hours,^20,21,44^ 3 studies reported outcome measures at 96 hours,^2,3,15^ while others reported outcome measures at 18 hours,^18^ 63 hours,^42^ and 72 hours^6^ postactivity.

The Kmet scores for included studies ranged from 77% to 100%, which represented good to strong methodological quality (Table S4). All included studies scored positively for the following Kmet criteria: design evident and appropriate to answer the study question; outcome measures well defined and robust to measurement and means of assessment reported; analysis described and appropriate; estimate of variance reported; and results support the conclusions for the main results/outcomes. Only one study received a partial score for insufficient detail reported in its Results section.^42^ In regard to sufficient description of participant characteristics, appropriate sample size, and confounding control, 86%, 57%, and 50% of the studies, respectively, scored positively, with the remainder scoring partial results (Table S4). Only one study received a positive score for the appropriate method of participant selection and description^18^ (Table S4).

### Quantitative Analyses

**FIGURE 1.**
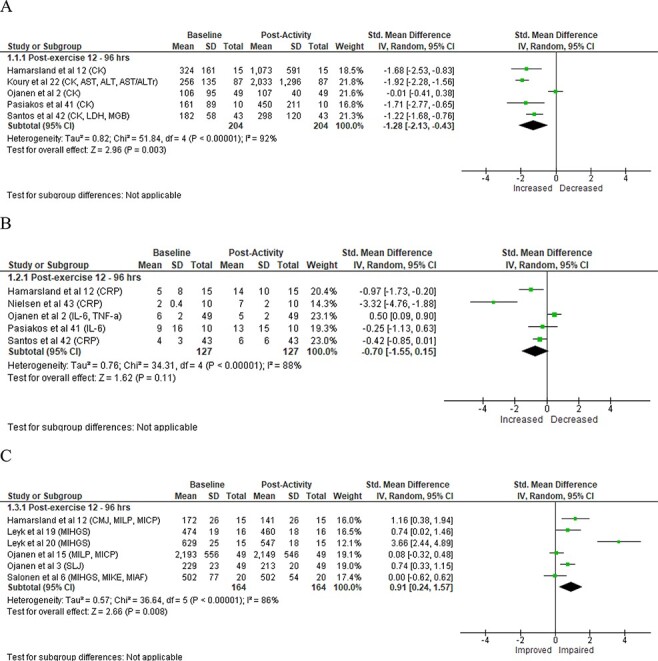
Forest plot of muscle damage, inflammation, and strength and power. Forest plots of indirect markers of muscle damage with standardized (Std.) mean differences and associated 95% confidence intervals (CI) for (A) muscle damage (aspartate aminotransferase [AST], alanine aminotransferase [ALT], AST/ALT ratio [AST/ALTr], creatine kinase [CK], lactate dehydrogenase [LDH], and myoglobin [MGB]), (B) inflammation ( C-reactive protein [CRP], interleukin 6 [IL-6], and tumor necrosis factor alpha [TNF-a]), and (C) strength and power (counter-movement jump [CMJ], maximal isometric arm flexion [MIAF], maximal isometric chest press [MICP], maximal isometric hand grip strength [MIHGS], maximal isometric knee extension [MIKE], maximal isometric leg press [MILP], and standing long jump [SLJ].

**FIGURE 2.**
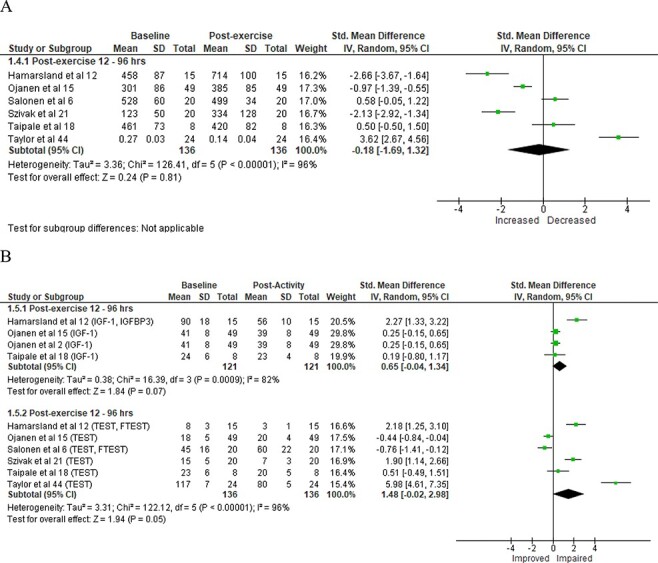
Forest plot of hormonal markers. Forest plots of hormonal responses with standardized (Std.) mean differences and associated 95% confidence intervals (CI) for (A) Cortisol and (B) anabolic hormones (insulin growth factor 1 [IGF-1], insulin growth factor binding protein 3 [IGFBP3], testosterone [TEST], and free testosterone [FTEST]).

For biomarkers of indirect muscle damage, data were extracted from 5 studies with muscle damage significantly greater postactivity compared to preactivity measures (Fig. 1A) and a large magnitude of difference. Data for the markers of inflammation were extracted from 5 studies with no significant changes observed postactivity compared to preactivity (Fig. 1B) with a moderate magnitude of difference. For the strength and power assessments, data were extracted from 6 studies with significant impairments in outcome measures postactivity compared to preactivity (Fig. 1C) and a large magnitude of difference. No significant changes were observed for CORT levels postactivity when compared to preactivity measures (Fig. 2A). Similarly, no significant postactivity changes were noted for IGF-1 with a moderate effect size (Fig. 2B). In contrast, TEST was significantly impaired at postactivity when compared to preactivity measures with a large magnitude of difference (Fig. 2B). While differences were noted for some outcome measures, the *I*^2^ score values for studies ranged from 82% to 96%, indicating high heterogeneity for all outcome measures.

## DISCUSSION

The current systematic review examined the acute effects of various military-specific physical training on biomarkers of exercise-induced stress and various strength and power measures. The meta-analysis showed that military-specific training significantly increased markers of muscle damage with no changes in inflammatory markers, whilst strength and power outcome measures were impaired. For the endocrinological responses, no differences were observed for cortisol or IGF-1, while TEST was significantly decreased postexercise. Therefore, these findings suggest that military-specific physical training significantly impacted physical performance and fatigue with variable responses based on different population groups, training protocols and environments, and evaluation methods. Furthermore, military training resulted in significant physiological and performance changes, with these parameters likely to be the most appropriate outcome measures to adequately monitor the response.

The current meta-analysis supports previous findings of physical training-induced reductions in strength and power assessments in military personnel,^8,23^ with these remaining reduced for up to 96 hours.^3,12,15,19,20^ This impairment may result from a failure of excitation–contraction coupling, limited muscle perfusion, oxygen supply and removal of metabolites, and attenuated muscular contractility,^20^ leading to fatigue.^45,46^ The observed impairment of strength and power may also result from significant mechanical damage to skeletal muscle,^47^ with increases in indirect markers (e.g., CK) identified postactivities (Fig. 1). Many of the military-based activities examined in this meta-analysis included eccentric actions (e.g., jumping and sprinting), a known precursor to significant muscle damage.^47^ Subsequently, this reduced performance could impact personnel directly during occupational tasks and lead to an inability to move across the terrain in a tactical environment, inability to negotiate obstacles, and poor marksmanship.^25,26^ Furthermore, these performance reductions may compromise the quality of subsequent military training, resulting in sub-optimal adaptations^27^ and/or increased risk of injury.^28^ Therefore, strength and power assessments may provide an easy-to-use, low-cost method for commanders and PT staff to evaluate muscle damage, fatigue, and performance impairment and training adaptations in military personnel. For example, regular strength and power testing, such as repetition maximums and/or CMJs, are low-cost and practical assessments that can easily be implemented by the military PT staff. The results of which could be used to inform commanders of their personnel’s level of readiness, prior to undertaking further training.

As previously identified, significant muscle damage occurs following military training with an inflammatory response expected, underpinning the potential mechanisms of impairment in muscular contractility.^32^ In prior studies, muscle damage and inflammatory responses were reported to follow similar changes following military activities.^2,12^ However, the current meta-analysis showed no significant increase in markers of inflammation (Fig. 1). This minimal inflammatory effect may reflect the methodology of current studies rather than the true effect of the activity, as reductions of activity prior to blood sampling^2,42^ and a limited activity duration^41^ may have seen markers of inflammation return to baseline levels prior to postactivity testing. Therefore, while it has been suggested that monitoring levels of inflammatory markers may also assist the evaluation of military personnel’s physical strain during physical preparation,^24^ caution is required when interpreting the results. Future studies are encouraged to examine markers of inflammation near the completion of PT in order to determine their suitability to monitor acute and longer-term responses.

The meta-analysis showed mixed hormonal responses after various military training activities, with TEST significantly lower and IGF-1 demonstrating a nonsignificant moderate effect after activities (Fig. 2). Reductions in anabolic hormones like TEST and IGF-1 have been shown to be a result of physical strain and energy/sleep deficits,^6,15^ common components of military training.^8^ This reduction in anabolic hormones may indirectly lead to a greater catabolic effect in soldiers with lower ability to repair muscle damage and recover from strenuous physical activity.^15^ However, no significant changes in CORT were noted in the current meta-analysis. The meta-analysis results for CORT were variable between studies (Fig. 2) with a lack of a significant overall change in CORT possibly influenced by 3 studies due to less physically demanding tasks prior to sampling collection,^44^ duration/intensity of the activity,^18^ and recovery period prior to sampling collection.^6^ Future studies should examine CORT samples immediately after the completion of physical activity to optimize the sampling procedure and document the authentic stress response. Additionally, like inflammatory markers, monitoring of TEST throughout training cycles may assist fatigue management, given that appropriate recovery is required following physically demanding training for optimal adaptations.^48^ For example, salivary measures of CORT or TEST are easily collected with commercially available biochemical analyzers and thus may be used posttraining to monitor personnel’s fatigue status and ensure a return to baseline before commencing subsequent strenuous activities.

Interestingly, apart from CORT, the responses observed for each measure were generally consistent and independent of training modality. For example, measures of strength and power, muscle damage, and TEST were significantly impaired following FTX-,^2,3,12,15,42^ PT-,^18–20,41^ and SERE-based^21,44^ activities. While not significant, IGF-1 and measures of inflammation displayed similar negative responses regardless of training modality. Cortisol was the only examined measure that demonstrated a variable result with training modality. This response was largely due to the extreme and opposite changes reported by Taylor et al.^44^ and Szivak et al.^21^ for CORT at 24 hours post-SERE training. Both studies investigated similar participants over similar time frames and activities; however, Szivak et al.^21^ utilized blood drawn samples, whereas Taylor et al.^44^ utilized salivary samples. Subsequently, differences in sampling methods may be an important factor to consider for postexercise biomarker assessments that should be considered in future research.

Several limitations of the included studies should be acknowledged. First, only 3.5% of participants were female, which prevented a separate analysis given the small numbers. With the inclusion of females in a greater number of roles in the military, particularly combat roles, it would be beneficial for future research to examine sex differences. Different baseline physical fitness levels and hormonal levels between sexes may potentially impact recovery kinematics,^49^ with future studies to confirm such sex influences. A further limitation was that the majority of biomarkers and/or physical performance measures were examined acutely (18–24 hours) postexercise,^18,20,21,44^ when it is clear that activity-induced stress can continue for several days postexercise.^2,3,12,15,41^

A number of limitations should be addressed for the current systematic review. First, different measures of strength and power assessments, markers of muscle damage, hormonal changes, and inflammation were examined in the review, with each potentially having varying recovery properties. Thus, it is possible that the distinct factors of each variable may have influenced the overall results of the meta-analysis. Second, within each outcome measure, individual components of each could not be separated due to the limited number of studies. This pooling of outcome measures may challenge the ability to find a true meaningful difference, given the variation in clinical significance and sensitivity of the different measures. However, the review does provide guidance for researchers as to the relevant monitoring tools to use following military-specific training in future research. Third, the sample sizes of studies were small to modest (8–87 participants), with a high level of heterogeneity of participants. These aspects were not examined in detail in the current review, with the factors causing differences among the studies requiring future work.^50^ Finally, there was a great deal of variation in the length of the interventions, participant demographics, recovery times, activities undertaken, and outcome measures examined with future studies to clarify these impacts further.

An important and novel strength of this systematic review was that it examined the physiological response and recovery periods of a broad range of military activities via meta-analysis, unlike previous reviews that provided overviews and summaries of common markers of fatigue in military training.^8,23,24^ As a result, the transferability of the findings to training regimes within the military is broader in scope. A further strength of this study was our incorporation of biomarkers of indirect muscle damage, inflammation, and anabolic/catabolic hormones. The examination of these biomarkers may help provide details of the potential mechanisms underpinning impairment in physical performance and areas warranting further research.^24^ The final strength of this review was the focus on strength and power measures, important contributors to military performance.^4^ However, physical assessments in other fitness domains (e.g., aerobic and endurance) may need to be examined in the future to determine their validity as tools to monitor fatigue in military personnel.

## CONCLUSION

The modern battlefield provides many challenges for the soldier’s physical capacity with rigorous physical occupational training needed to prepare the soldier. This systematic review and meta-analysis indicated that assessments of strength, power, and hormonal levels may provide early indications of physiological fatigue and/or training adaptations. Regular use of these outcomes by military PT and command staff may enable enhanced monitoring of physiological stress and training regimes to optimize adaptations for the soldier’s occupational performance.

## Supplementary Material

usac144_Supp
