# Supplementary material for: Acute Fatigue Responses to Occupational Training in Military Personnel: A Systematic Review and Meta-Analysis
Source: Mil Med. 2022 May 27;188(5-6):969–77. doi: 10.1093/milmed/usac144 (PMC10187475; doi:10.1093/milmed/usac144)
Supplement: usac144_Supp [file usac144_supp.zip › Supplementary Table 2..pdf]

Supplementary Table 2. Participant characteristics and methodological quality of included studies.

Study	*Sex*: sample size	Participant demographics	Military group	Limitations of each study as per Kmet criteria	Overall methodological quality via Kmet
Hamarsland et al.[12]	*Male:* 15	Age: 23 ± 4 y<br>Mass: 78 ± 7 kg<br>Height: 181 ± 6 cm	Apprentices participating in an annual selection course to join the Norwegian Naval Special Forces	Partial description of method of subject selection<br>Insufficient data to assess appropriateness of sample size<br>Incomplete control of confounding	Strong
Koury et al.[22]	*Male:* 87	Age: 20 ± 2 y<br>Mass: 72.5 ± 7.9 kg<br>Height: *not reported*	Brazilian Army cadets	Partial description of method of subject selection<br>Insufficient data to assess appropriateness of sample size<br>Incomplete control of confounding	Strong
Leyk et al.[19]	*Male:* 17<br>*Female:* 15	*Male:* Age: 27.1 ± 9.4 y<br>Mass: 81.1 ± 10.6 kg<br>Height: 179 ± 6 cm<br>*Female:* Age: 29.8 ± 7.4 y<br>Mass: 65.7 ± 12.1 kg<br>Height: 167 ± 7 cm	Members of the Medical Services of the German Armed Forces	Partial description of method of subject selection	Strong
Leyk et al.[20]	*Male:* 15	Age: 23.9 ± 6.1 y<br>Mass: 79.1 ± 12.7 kg<br>Height: 180 ± 6 cm	Members of the Medical Services of the German Armed Forces	Partial description of method of subject selection	Strong
Nielsen et al.[43]	*Male:* 10	Age: 21 - 28 y (only range reported)<br>Mass: *not reported*<br>Height: *not reported*	Soldiers participating in the Norwegian Ranger-training course	Question or objective partially described<br>Partial description of method of subject selection<br>Partial description of subject characteristics<br>Insufficient data to assess appropriateness of sample size<br>Incomplete control of confounding	Good
Ojanen et al.[3]	*Male: 49*	Age: 20 ± 1 y<br>Mass: 73.5 ± 8.7 kg<br>Height: 179 ± 6 cm	Finnish Army conscripts undertaking compulsory service	Partial description of method of subject selection	Strong

Study	*Sex*: sample size	Participant demographics	Military group	Limitations of each study as per Kmet criteria	Overall methodological quality via Kmet
Ojanen et al.[2]	*Male: 49*	Age: 20 ± 1 y<br>Mass: 73.5 ± 8.7 kg<br>Height: 178.5 ± 6.4 cm	Finnish Army conscripts undertaking compulsory service	Partial description of method of subject selection<br>Insufficient data to assess appropriateness of sample size	Strong
Ojanen et al.[15]	*Male: 49*	Age: 20 ± 1 y<br>Mass: 73.5 ± 8.7 kg<br>Height: 179 ± 6 cm	Finnish Army conscripts undertaking compulsory service	Partial description of method of subject selection	Strong
Pasiakos et al.[41]	*10* (Genders not given for experimental groups)	Age: 24 ± 5 y<br>Mass: 77 ± 10 kg<br>Height: 177 ± 8 cm	U.S. Military personnel, Human Research Volunteer recruit platoon, and civilians from the local area.	Partial description of method of subject selection<br>Insufficient data to assess appropriateness of sample size	Strong
Salonen et al.[6]	*Male:* 20	Age: 20 ± 1 y<br>Mass: 76 ± 7.1 kg<br>Height: *not reported*	Reconnaissance conscripts	Partial description of method of subject selection Partial description of subject characteristics Incomplete control of confounding	Strong
Santos et al.[42]	*Male:* 43	Age: 19 - 24 y (only range reported)<br>Mass: 74.4 ± 7.7 kg<br>Height: *not reported*	Brazilian Military Recruits	Partial description of method of subject selection<br>Insufficient data to assess appropriateness of sample size<br>Incomplete control of confounding<br>Results reported in insufficient detail	Strong
Szivak et al.[21]	*Male:* 20	*High fit group (n = 10):*<br>Age: 25.3 ± 4.39 y<br>Mass: 82.21 ± 17.85 kg<br>Height: 176.22 ± 10.81 cm<br>*Low fit group (n = 10):*<br>Age: 25.2 ± 9.02 y<br>Mass: 85.24 ± 30.40 kg<br>Height: 180 ± 12.24 cm	Active members of the U.S. Navy and Marine Corps.	Partial description of method of subject selection Incomplete control of confounding	Strong

Study	*Sex*: sample size	Participant demographics	Military group	Limitations of each study as per Kmet criteria	Overall methodological quality via Kmet
Taipale et al.[18]	*Male:* 8	Age: 27 ± 4 y<br>Mass: 72 ± 11 kg<br>Height: 180 ± 8 cm	Finnish Defence Force Reservists	Partial description of method of subject selection	Strong
Taylor et al.[44]	*Male:* 24	Age: 27.8 ± 6 y<br>Mass: *not reported*<br>Height: *not reported*<br>BMI: 29 ± 4.7 kg/m^2	Active duty Navy and Marine Corps personnel undertaking SERE training	Partial description of method of subject selection Incomplete control of confounding	Strong
