# Supplementary material for: Acute Fatigue Responses to Occupational Training in Military Personnel: A Systematic Review and Meta-Analysis
Source: Mil Med. 2022 May 27;188(5-6):969–77. doi: 10.1093/milmed/usac144 (PMC10187475; doi:10.1093/milmed/usac144)
Supplement: usac144_Supp [file usac144_supp.zip › Supplementary Table 3. .pdf]

**Supplementary Table 3.** Methodological description and general findings of included studies.

| Study | Training intervention | Activity type and duration | Recovery time points analysed | Outcome measures | Results |
|---|---|---|---|---|---|
| Hamarsland et al.[12] | A so-called "hell week", which consisted of sleep and caloric restriction and extreme amounts of physical activity for 20-hours a day, in a very stressful environment | One week – FTX* | 24- and 72-hours and 1 and 2 weeks after the "hell week" | CMJ*, Maximal isometric leg press, maximal isometric chest press, CK*, $TEST^T$*, $TEST^F$*, CORT*, CRP* T/C Ratio*, IGF-1*, IGFBP3*, T3*, T4*, TSH*, SHBG* | Significant reduction (*P*<0.05) of CMJ at all time points and leg press and chest press at 24-, 72-hours and 1 week compared to baseline. $TEST^F$ was significant decreased (*P*<0.05) at 24-, 72-hours and 1 week compared to baseline. $TEST^T$ and IGFBP3 were significantly lower (*P*<0.05) at 24- and 72-hours, as was T/C Ratio, IGF-1 and T3 at 72-hours, compared to baseline. T4 was also significantly lower (*P*<0.05) at 1 week, compared to baseline. SHBG, CK and CRP were all significantly increased (*P*<0.05) at 24-hours. SHGB and CORT were significantly increased (*P*<0.05) at 72-hours and $TEST^T$, CORT and TSH were significantly higher (*P*<0.05) than base line after 1 week recovery. |
| Koury et al.[22] | A traditional army physical training program consisting of a 30-km march at 4 km/h followed by a 2-km field march while carrying loads on day 1. Uninterrupted military physical training occurred on the second and third days | 3 Days – FTX | 12-hours after the 30 km march and 48-hours after military training | CK, ALT*, AST*, AST/ALT Ratio | Significantly greater (*P*<0.001) serum concentrations of all markers at both time points compared to baseline |

| Study | Training intervention | Activity type and duration | Recovery time points analysed | Outcome measures | Results |
|---|---|---|---|---|---|
| Leyk et al.[19] | 50 kg Stretcher carriage with both hands on a treadmill at a speed of 4.5 km/h, until exhaustion | Until exhaustion (approx 64 - 235 seconds) – PT* | 24-, 48- and 72-hours after the load carriage task | Maximal isometric hand-grip strength | Significantly reduced ($P$<0.05) hand-grip forces in males at 24-hours compared to baseline |
| Leyk et al.[20] | 50 kg Stretcher carriage with both hands on a treadmill at a speed of 4.5 km/h, until exhaustion | Until exhaustion (approx 128 - 302 seconds) – PT | 24-hours after the load carriage task | Maximal isometric hand-grip strength | Significantly reduced ($P$<0.05) hand-grip forces at 24-hours compared to baseline |
| Nielsen et al.[43] | The ranger-training course consisted of semicontinuous physical activity including patrols during night-time, and, during the day, combat training with attacks, building defence positions, and passing obstacles and narrow tubes containing water. No food was provided during the course and sleep was limited to 3-hours a day | 8-days – FTX | 24- and 72-hours after the ranger-training course | CRP | Nil significant changes at either recovery point compared to baseline |
| Ojanen et al.[3] | Military field training including combat drills and live fire shooting exercises, reconnaissance, combat manoeuvres, patrolling, tactical road marches and full combat mission profiles | 21-days – FTX | 96-hours after the military field training | SLJ*, 60-second push-up test, 60-second sit-up test, RPE* | Significant reduction ($P$<0.01) in all measures compared to baseline |

| Study | Training intervention | Activity type and duration | Recovery time points analysed | Outcome measures | Results |
|---|---|---|---|---|---|
| Ojanen et al.[2] | Military field training including combat drills and live fire shooting exercises, reconnaissance, combat manoeuvres, patrolling, tactical road marches and full combat mission profiles | 21-days – FTX | 96-hours after the military field training | IGF-1, IL-6*, Leptin, TNF-*a*, CK, SLJ, 60-second push-up test, 60-second sit-up test, RPE | Significant decrease ($P$<0.05) in TNF-*a* compared to baseline |
| Ojanen et al.[15] | Military field training including combat drills and live fire shooting exercises, reconnaissance, combat manoeuvres, patrolling, tactical road marches and full combat mission profiles | 21-days – FTX | 96-hours after the military field training | CORT, $TEST^T$, maximal isometric leg press, maximal isometric bench press, SHBG, IGF-1 | Significant increase ($P$<0.05) in CORT and $TEST^T$, and a significant decrease ($P$<0.05) in maximal isometric bench press compared to baseline |
| Pasiakos et al.[41] | Load carriage on a treadmill wearing a weighted vest equivalent to 30% of body weight | 90-minutes – PT | 3-, 24-, 48- and 72-hours after the training intervention | CK, IL-6, hepciden, ferritin | Significant increase ($P$<0.05) in CK at all time points, ferritin at 24-, 48- and 72-hours, and hepciden at 3- and 24-hours, compared to baseline. |
| Salonen et al.[6] | Garrison training (4-days) included lectures, military skill training and physical training and military field training (8-days) focused on rehearsing the basic skills of a reconnaissance soldier and rehearsals conducting reconnaissance during offensive operations | 12-days – PT, MST* & FTX | 72-hours after the military field training | CORT, $TEST^F$, $TEST^T$, T/C ratio, SHBG, T4, hand grip strength, maximal isometric contraction of knee extensors and arm flexors (pre-done after 5-days training and in only 10 participants) | Significant increase in SHBG ($P$<0.05) and $TEST^F$ ($P$<0.01) compared to baseline |

| Study | Training intervention | Activity type and duration | Recovery time points analysed | Outcome measures | Results |
|---|---|---|---|---|---|
| Santos et al.[42] | 24-hour continuous operations, carrying a 25kg load carried, over 25 km per day | 4-days – FTX | 63-hours after the training activity | MGB*, LDH*, CRP, CK, RPE | Significant increase ($P$<0.001) in LDH compared to baseline |
| Szivak et al.[21] | Navy SERE* training including a 4 day didactic phase, followed by field training phase including practicing evasion techniques, traversing several miles of steep snow-covered terrain while carrying a military-issue rucksack, and a final capture phase culminating in several high stress training scenarios designed to provide realistic captivity experiences | 2-weeks – MST & FTX | 24-hours post SERE training | CORT, TEST[T], epinephrine, norepinephrine, dopamine and NPY* | Significant increase ($P$<0.05) in CORT, norepinephrine, dopamine and NPY and a significant decrease ($P$<0.05) in TEST[T] compared to baseline |
| Taipale et al.[18] | Loaded marching (on treadmill) wearing a rucksack, a helmet, an unloaded/ inoperable rifle, and army boots for a load of approximately 16 kg | 50-minutes – PT | 18-hours post training activity | CORT, TEST[T] Maximal isometric force of the leg extensors, SHBG, IGF-1, | Nil significant changes compared to baseline |

| Study | Training intervention | Activity type and duration | Recovery time points analysed | Outcome measures | Results |
|---|---|---|---|---|---|
| Taylor et al.[44] | SERE training including a 5-day didactic training phase, followed by applied training in survival, evasion, resistance, and escape techniques. This was followed by a field phase where participants were required to evade 'enemy' captors. Upon eventual capture, participants apply their skills of resistance to political indoctrination and captivity-related challenges | 12-days – MST & FTX | 24-hours post SERE training | CORT, $TEST^T$, DHEA, DHEA-S | A main time effect was evident for a group that ingested DHEA and placebo, with testosterone significantly lower, whilst cortisol was significantly higher ($P<0.05$) during the activity, but lower at 24-hours post-stress ($P<0.05$). |

*ALT* alanine aminotransferase, *AST* aspartate aminotransferase, *CMJ* countermovement jump, *CORT* cortisol, *CK* creatine kinase, *CRP* C-reactive protein, *DHEA* dehydroepiandrosterone, *DHEA-S* dehydroepiandrosterone sulfate, *IL-6* interleukin 6, *IGFBP3* insulin-like growth factor binding protein 3, *IGF-1* insulin-like growth factor one, *MST* military skills training, *MGB* myoglobin, *LDH* lactate dehydrogenase, *PSS-10* perceived stress scale-10 item, *PT* Physical training, *NPY* neuropeptide Y, *RPE* rate of perceived exertion, *SHBG* sex hormone binding globulin, *SLJ* standing long jump, *T3* triiodothyronine, *T4* thyroxine, *T/C Ratio* testosterone to cortisol ratio, $TEST^F$ free testosterone, $TEST^T$ total testosterone, *TNF-a* tumour necrosis factor alpha, *TSH* thyroid-stimulating hormone, *FTX* field training exercise
