# Supplementary material for: Acute Fatigue Responses to Occupational Training in Military Personnel: A Systematic Review and Meta-Analysis
Source: Mil Med. 2022 May 27;188(5-6):969–77. doi: 10.1093/milmed/usac144 (PMC10187475; doi:10.1093/milmed/usac144)
Supplement: usac144_Supp [file usac144_supp.zip › Supplementary Table 4..pdf]

Supplementary Table 4. Kmet ratings of all included studies.

	1	2	3	4	8	9	10	11	12	13	14	Rating (%)
Hamarsland et al.[12]	2	2	1	2	2	1	2	2	1	2	2	86[a]
Koury et al.[22]	2	2	1	2	2	1	2	2	1	2	2	86[a]
Leyk et al.[19]	2	2	1	2	2	2	2	2	2	2	2	95[a]
Leyk et al.[20]	2	2	1	2	2	2	2	2	2	2	2	95[a]
Nielsen et al.[43]	1	2	1	1	2	1	2	2	1	2	2	77[b]
Ojanen et al.[3]	2	2	1	2	2	2	2	2	2	2	2	95[a]
Ojanen et al.[2]	2	2	1	2	2	1	2	2	2	2	2	91[a]
Ojanen et al.[15]	2	2	1	2	2	2	2	2	2	2	2	95[a]
Pasiakos et al.[41]	2	2	1	2	2	1	2	2	2	2	2	91[a]
Salonen et al.[6]	2	2	1	1	2	2	2	2	1	2	2	86[a]
Santos et al.[42]	2	2	1	2	2	1	2	2	1	1	2	82[a]
Szivak et al.[21]	2	2	1	2	2	2	2	2	1	2	2	91[a]
Taipale et al.[18]	2	2	2	2	2	2	2	2	2	2	2	100[a]
Taylor et al.[44]	2	2	1	2	2	2	2	2	1	2	2	91[a]
								Median rating				91

[a]Strong quality; [b]Good quality; Questions 5-7 were inapplicable due to the methodological design of included studies and removed from the Kmet appraisal.
