# Supplementary material for: Acute Fatigue Responses to Occupational Training in Military Personnel: A Systematic Review and Meta-Analysis
Source: Mil Med. 2022 May 27;188(5-6):969–77. doi: 10.1093/milmed/usac144 (PMC10187475; doi:10.1093/milmed/usac144)
Supplement: usac144_Supp [file usac144_supp.zip › Supplementery Table 1. Search strategy.pdf]

Supplementary Table 1. Search strategy

**Initial search: Assessment retrieval**	**Database and Search Terms**	**Limitations**
**Subject Headings**	**PubMed:** (Human [Mesh] OR adult [Mesh]) AND (military personnel [Mesh] OR military medicine [Mesh]) AND (exercise test [Mesh] OR exercise [Mesh] OR task performance and analysis [Mesh] OR physical exertion [Mesh] OR physical fitness [Mesh] OR physical endurance [Mesh] OR biomarkers [Mesh] OR creatine kinase [Mesh] OR hormones [Mesh] OR catecholamines [Mesh] OR cytokines [Mesh] OR growth hormone [Mesh] OR biomarkers [Mesh] OR hydrocortisone [Mesh] OR saliva [Mesh] OR testosterone [Mesh] OR insulin-like growth factor binding protein (1-6) [Mesh] OR sex hormone-binding globulin [Mesh] OR amylase [Mesh])	Humans; English; Adult: 19+ years
**Free Text Words**	**CINAHL*:*** (military OR army OR “air force”) AND (cortisol OR "Creatine kinase" OR inflamm* OR soreness OR "Oxidative stress") AND (training OR combat OR (load AND carriage) OR task)	Publication date from 2000 – current; Adult: 18 to 64 years OR Aged: 65+ years
	**PubMed:** *As per CINHAL Free Text*	Publication date from 2000 to 29/06/2021; Humans; English; Adult: 19+ years
	**Scopus:** *As per CINHAL Free Text*	*As per CINHAL Free Text*
	**SportDiscus:** *As per CINHAL Free Text*	*As per CINHAL Free Text*
	**Web of Science:** *As per CINHAL Free Text*	*As per CINHAL Free Text*
