# Supplementary figures and images for: Acute Fatigue Responses to Occupational Training in Military Personnel: A Systematic Review and Meta-Analysis
Source: Mil Med. 2022 May 27;188(5-6):969–77. doi: 10.1093/milmed/usac144 (PMC10187475; doi:10.1093/milmed/usac144)

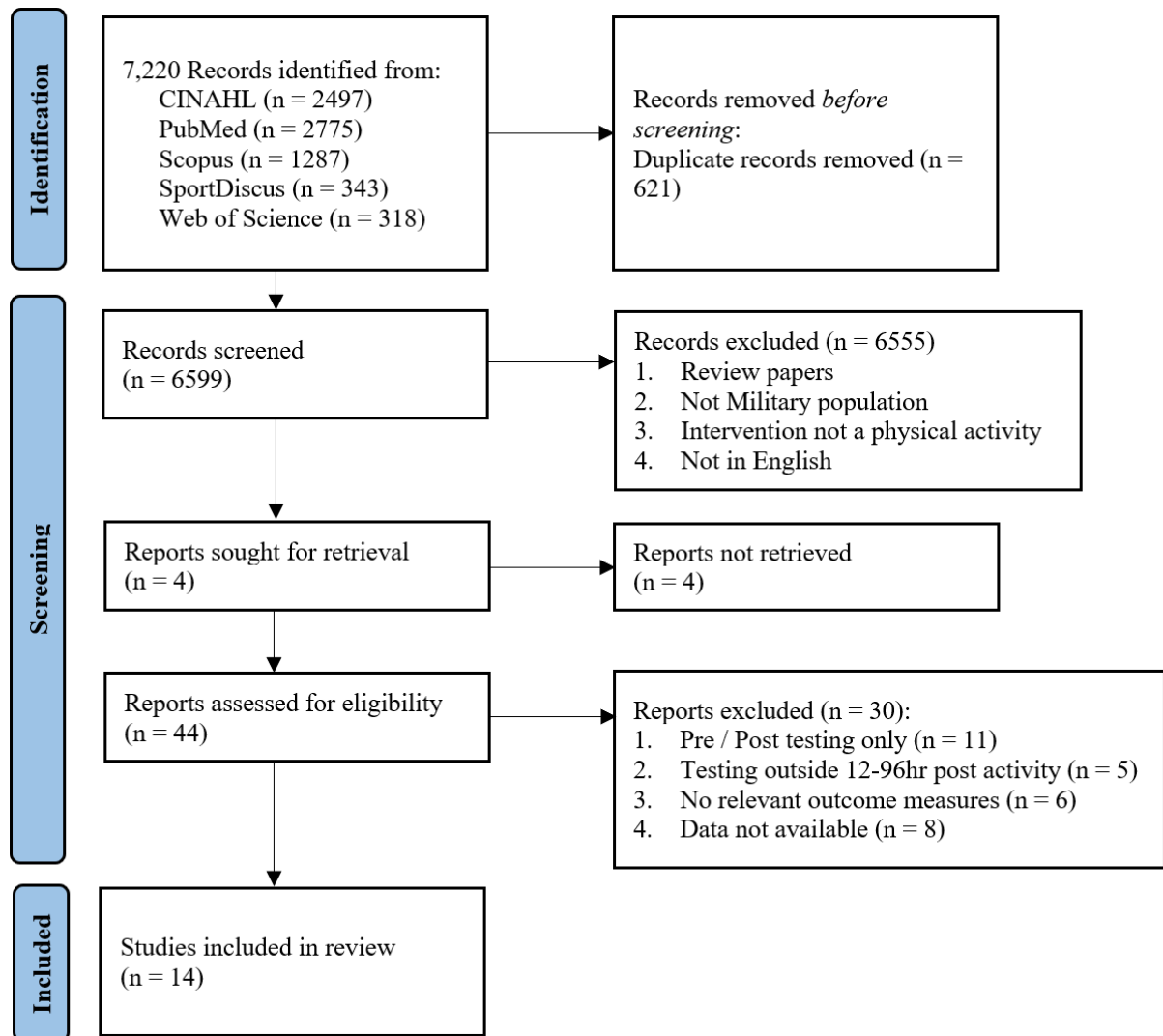

**Supplementary Figure 1.** Flowchart of the search strategy according to the PRISMA guidelines.

Supplement: usac144_Supp [file usac144_supp.zip › Supplementery Figure 1.pdf]
